# Response to Malnutrition Treatment in Low Weight-for-Age Children: Secondary Analyses of Children 6–59 Months in the ComPAS Cluster Randomized Controlled Trial

**DOI:** 10.3390/nu13041054

**Published:** 2021-03-24

**Authors:** Jeanette Bailey, Natasha Lelijveld, Tanya Khara, Carmel Dolan, Heather Stobaugh, Kate Sadler, Richard Lino Lako, André Briend, Charles Opondo, Marko Kerac, Mark Myatt

**Affiliations:** 1 International Rescue Committee, New York, NY 10168, USA; 2 Department of Population Health, London School of Hygiene & Tropical Medicine, London WC1E 7HT, UK; marko.kerac@lshtm.ac.uk; 3 Emergency Nutrition Network, Oxford OX5 2DN, UK; natasha.lelijveld.11@alumni.ucl.ac.uk (N.L.); tanya@ennonline.net (T.K.); carmel@n4d.group (C.D.); kate@ennonline.net (K.S.); 4 Action Against Hunger, New York, NY 10004, USA; hstobaugh@actionagainsthunger.org; 5 Department of Policy, Planning, Budgeting and Research, Ministry of Health, Juba, South Sudan; richardlako@yahoo.com; 6 Center for Child Health Research, Faculty of Medicine and Health Technology, Tampere University, FI-33014 Tampere, Finland; andre.briend@gmail.com; 7 Department of Nutrition, Exercise and Sports, University of Copenhagen, DK-2200 Copenhagen, Denmark; 8 Department of Medical Statistics, London School of Hygiene & Tropical Medicine, London WC1E 7HT, UK; charles.opondo@lshtm.ac.uk; 9 Centre for Maternal, Adolescent, Reproductive, & Child Health, London School of Hygiene & Tropical Medicine, London WC1E 7HT, UK; 10 Brixton Health, Llwyngwril, Gwynedd LL37 2JD, Wales, UK; mark@brixtonhealth.com

**Keywords:** acute malnutrition, weight-for-age, mid-upper arm circumference, ready-to-use therapeutic food, community-based management of acute malnutrition, wasting, stunting, concurrent wasting and stunting

## Abstract

Weight-for-age z-score (WAZ) is not currently an admission criterion to therapeutic feeding programs, and children with low WAZ at high risk of mortality may not be admitted. We conducted a secondary analysis of RCT data to assess response to treatment according to WAZ and mid-upper arm circumference (MUAC) and type of feeding protocol given: a simplified, combined protocol for severe and moderate acute malnutrition (SAM and MAM) vs. standard care that treats SAM and MAM, separately. Children with a moderately low MUAC (11.5–12.5 cm) and a severely low WAZ (<−3) respond similarly to treatment in terms of both weight and MUAC gain on either 2092 kJ (500 kcal)/day of therapeutic or supplementary food. Children with a severely low MUAC (<11.5 cm), with/without a severely low WAZ (<−3), have similar recovery with the combined protocol or standard treatment, though WAZ gain may be slower in the combined protocol. A limitation is this analysis was not powered for these sub-groups specifically. Adding WAZ < −3 as an admission criterion for therapeutic feeding programs admitting children with MUAC and/or oedema may help programs target high-risk children who can benefit from treatment. Future work should evaluate the optimal treatment protocol for children with a MUAC < 11.5 and/or WAZ < −3.0.

## 1. Introduction

Malnutrition is a major underlying cause of morbidity and mortality in children [1]. At any given time, stunting, defined by deficits in a child’s height-for-age, affects at least 149 million children under five years of age, and wasting, defined by deficits in a child’s weight-for-height and/or a low mid-upper arm circumference, affects about 49 million children [2]. The incidence of wasting in children may be far higher than these prevalence estimates suggest [3,4]. Malnutrition contributes to approximately half of all deaths in children less than 5 years of age [1], and deaths associated with wasting may also be significantly underestimated [5]. The coronavirus pandemic is likely to further increase wasting and associated mortality [6].

Current policy and practice organize programs that focus on stunting and wasting separately [7,8,9,10]. Stunting is typically addressed through development programs that primarily focus on its prevention, whereas wasting is often addressed through humanitarian programs that primarily focus on its treatment in order to prevent near-term mortality. Multisectoral social and development programs aim to prevent stunting through agriculture; micronutrient supplementation; infant and young child feeding; and water, sanitation, and hygiene (WASH) approaches, among others [11]. The management of wasting may include therapeutic and supplementary feeding and treatment of underlying illnesses. Despite evidence of the common causal pathways for wasting and stunting [12,13] and the epidemiological overlap [14], programs rarely integrate objectives to address both forms of undernutrition simultaneously. Current funding mechanisms and organizational structures facilitate this division, with grants usually directed to programs addressing either stunting or wasting but seldom both [7]. In practice, this means programs addressing both wasting and stunting may not be available at the same place and time. Similar silos exist within wasting treatment, as severe and moderate acute malnutrition (SAM and MAM) are treated in separate programs [7,15].

Children with concurrent wasting and stunting are among the most vulnerable of all malnourished children, with a higher mortality risk than either wasting or stunting alone, and about a 12 times greater risk of mortality in the absence of treatment than those with normal anthropometry [16,17]. In low-income countries, 4.7% of children are affected simultaneously by wasting and stunting [18]. Acknowledging the coexistence of wasting and stunting and the interactive effect on mortality risk, recent research has explored existing data to understand more about this particularly vulnerable group. Existing measures used at the community level were evaluated for their ability to identify concurrently wasted and stunted children. Weight-for-age (z-scores from the international reference median, WAZ) was found to have high (i.e., >90%) sensitivity and specificity for identifying children who are concurrently wasted and stunted across multiple settings [17]. In an analysis of cohort data from Senegal, different anthropometric criteria were examined for their ability to predict mortality. A combination of a severely low mid-upper arm circumference (MUAC < 11.5 cm) and a severely low WAZ (i.e., WAZ < −2.8) detected all near-term (i.e., occurring within 6 months of measurement) deaths associated with either a weight-for-height z-score (WHZ) < −3 and/or concurrent wasting and stunting (WHZ < −2 and height-for-age z-score (HAZ) < −2) [19,20]. MUAC is already well evidenced to identify those at high risk for mortality and is easy to use [21,22,23,24]. Used together, these measures may be both practical and effective as MUAC and WAZ are already used at the community level for screening and for growth monitoring and promotion.

Current therapeutic feeding programs use MUAC < 11.5 cm, WHZ < −3 and/or presence of oedema as independent admission criteria [25,26]. Some children who are severely wasted and concurrently stunted are included by these criteria, but moderately wasted children who are concurrently stunted may not be captured for therapeutic feeding by these admission criteria despite their having a similar near-term mortality risk to severely wasted children [16,17]. Adding WAZ to the admission criterion for therapeutic feeding could improve the ability of these programs to identify and treat malnourished children most at risk of dying [19]. The intensity of treatment required by this additional group of children and the impact of their inclusion on therapeutic program caseloads and workload has yet to be evaluated in a prospective trial. There is already growing interest to simplify protocols for wasting treatment to reduce the silos between SAM and MAM treatment and optimize the use of resources to identify and treat the most vulnerable children [27,28,29,30,31,32,33,34,35,36]. Combining SAM and MAM treatment into a single protocol provides a holistic continuum of care that may enable treatment before children become severely malnourished. This paper therefore draws on data from a randomized controlled trial that tested the effectiveness of a simplified, combined protocol for treatment of medically uncomplicated severe and moderate acute malnutrition in children aged between 6 and 59 months [27,35].

The aim of this analysis was to contribute evidence on the value of adding WAZ to therapeutic feeding program admission criteria. Our objectives were to (1) assess outcomes and response to treatment in children admitted with a MUAC < 11.5 cm as two separate groups—those with a WAZ < −3 and those with a WAZ ≥ −3—both of which would be included in current therapeutic feeding programs, as well as children with a moderately low MUAC (i.e., MUAC between 11.5 cm and 12.5 cm) and a WAZ < −3, who would be eligible for admission into supplementary feeding programs in many settings; (2) explore outcomes and response to treatment by dosage protocol: a simplified, MUAC-based dosage in a combined treatment program compared to the weight-based dosage offered in standard care.

## 2. Materials and Methods

This paper reports on a secondary analysis of the intention-to-treat dataset from the Combined Protocol for Acute Malnutrition Study (ComPAS). ComPAS was a cluster-randomized controlled trial conducted in Kenya and South Sudan from May 2017–August 2018 to evaluate whether a simplified, combined protocol was non-inferior to standard care for treatment of medically uncomplicated SAM and MAM in children 6–59 months. The trial found non-inferior recovery (76.3% in the combined protocol arm and 73.5% in the standard protocol arm, risk difference of 0.03, 95% CI −0.05 to 0.10, *p* = 0.52), with less ready-to-use food required for a child with severe malnutrition to reach full recovery in the combined protocol (122 versus 193 sachets) and improved cost-effectiveness (US$123 less per child recovered; US$918 in the combined protocol versus US$1041 in the standard protocol) [35]. The methods [27,37], scientific rationale [34] and results [35] from the ComPAS trial have been published previously.

This study was a secondary analysis of data from a trial conducted according to the guidelines laid down in the Declaration of Helsinki, and all procedures involving research study participants were approved by the ethics committees of the London School of Hygiene and Tropical Medicine (reference 11826), the Kenya Medical Research Institute (reference non-KEMRI 551), and the Ministry of Health in South Sudan (approved 21 November 2016). The analyses conducted for this paper comply with these ethical approvals. Written informed consent was obtained from the caretakers of all patients or by a witness who could attest to the caretaker’s verbal consent.

In standard treatment, SAM and MAM are treated in separate programs with separate food products [7,15]. SAM is treated with ready-to-use-therapeutic food (RUTF) based on weight, and MAM can be treated with one sachet of ready-to-use supplementary food (RUSF) per day (Table 1). In the ComPAS intervention arm, a simplified, combined protocol admitted children with both SAM and MAM for treatment in a unified program, with eligible children receiving RUTF according to their MUAC and/or oedema status (Table 1, Supplementary Table S1). A simple MUAC-based RUTF dosage protocol was used because of evidence showing that MUAC identifies children at high-risk of mortality and is easier to use than weight-for-height measures [21,22,23,24], and that changes in MUAC track changes in weight [34,38,39]. The simplified protocol provides a reduced dosage of RUTF for children with a MUAC < 11.5 cm who have an admission weight of ≥5 kg, as these children would have received 2.5 sachets/day under the standard protocol (Supplementary Table S2).

Clusters in the ComPAS trial were 24 health facilities stratified by country and randomized to deliver either the combined protocol or the standard protocol [27]. The dataset included children aged between 6 and 59 months at admission diagnosed with moderate or severe wasting and without medical complications in three urban sub-counties of Nairobi, Kenya (Embakasi North, Embakasi East and Embakasi West) and Aweil East, a rural, agro-pastoralist region of Northern Bahr El Ghazal state in South Sudan. Children presenting to any of the 24 health clinics participating in the study were eligible to enroll. Active case finding was also conducted to refer children with malnutrition. Participants in Kenya were invited to attend an appointment four-months post-discharge as part of a follow-up study.

An anonymized dataset using only key variables from the ComPAS trial was used for this analysis [40]. The variables included case ID, age, sex, date of visit, anthropometric measurements (MUAC, weight, height, WAZ, HAZ) and oedema at each visit, outcome (recovered, died, defaulted, transferred or early discharge), site code, and intervention code (standard or combined protocol). Morbidity, hospitalization, fat mass, and fat-free mass variables were used from the follow-up study dataset. The following categorizations and anthropometric cut-offs were used:

- Group 1: MUAC < 11.5 cm and WAZ ≥ −3;
- Group 2: MUAC between 11.5 cm and <12.5 cm and WAZ < −3;
- Group 3: MUAC < 11.5 cm and WAZ < −3;
- Group 4: MUAC between 11.5 cm and <12.5 cm and WAZ ≥ −3.0.

Groups 1–3 were the groups of interest in this analysis, and group 4 is presented for comparison. Groups 1 and 3 are currently included in therapeutic feeding programs for treatment of SAM because MUAC < 11.5 cm is a standard admission criterion. Groups 2 and 4 are eligible for supplementary feeding for treatment of MAM where provided under existing guidelines because MUAC is between 11.5 and <12.5 cm [25,26]. Groups 1 and 3 received RUTF at a fixed dose of 4184 kJ (1000 kcal) RUTF/day in the combined protocol and RUTF at a standard dose 836.8 kJ (200 kcal)/kg/day in the standard protocol. Groups 2 and 4 received 2092 kJ (500 kcal) RUTF per day in the combined protocol and 2092 kJ (500 kcal) RUSF per day in the standard protocol (Table 1) [27]. RUTF and RUSF are similar in their compositions, but there are some key differences, including the proportion of total protein derived from dairy. RUTF typically has 50% of protein derived from dairy, and RUSF may have 33% of protein derived from dairy [25].

For each patient group, we assessed outcomes following treatment, duration of stay, and velocity of weight and MUAC change from admission until discharge. Outcomes included recovery (MUAC of ≥12.5 cm and no oedema for two consecutive visits), defaulting (failure to attend for three consecutive visits), death (including deaths that occurred after defaulting), transfers to other facilities, transfers to inpatient care, non-response (i.e., still under treatment but without recovery at or before the 17th week of treatment, and defined as “non-recovered” in this analysis) and early discharges (i.e., discharges made in error by facility staff before meeting recovery criteria for two consecutive visits, or being discharged as non-responders before the 17th week time limit). These issues are described in detail in the main trial paper [35]. We use the term “non-recovered” instead of “non-response” in this paper because there are some children who gained weight or MUAC and thus did “respond” to treatment but ultimately do not reach the recovery criteria. All outcome categories in the analysis were mutually exclusive.

In Kenya only, caregivers of all study participants were asked to return for a follow-up visit at four months following discharge. The outcomes assessed at this visit were anthropometry (weight, height, MUAC, and oedema), recent history of illness, and body composition using bioelectrical impedance analysis (BIA) [41]. For the body composition analysis, raw impedance data were converted to fat and fat-free mass (FFM) values (kg) using a calibration equation derived from healthy children aged 3 to 18 months in The Gambia [42].

Because the data came from a cluster-randomized controlled trial, outcome analyses were adjusted for clustering of observations within health facilities using a cluster-robust estimator of variance. We used binomial regression for binary outcomes and linear regression for continuous outcomes. Adjusted analyses accounted for country, age, and sex. Analyses were conducted using Stata IC v.13.1 (StataCorp LLC: College Station, TX, USA). Stata’s zscore06 module was used to calculate weight-for-age z-scores and flags, using WHO 2006 Growth Standards [43]. Children with biologically implausible WAZ values as determined by WHO flagging criteria (WAZ > 5 or <−6) at admission were excluded [44,45], and children with oedema on admission were not included in this analysis because their weight-for-age on admission would be unduly high due to the oedema.

To develop individual growth curves, MUAC and weight-for-age were plotted at each visit and examined. One set of growth curves looks only at children who achieved recovery within 17 weeks, to allow for a closer examination of how these children recovered. CMAM programs typically report average weight and MUAC gains among recovered children only; therefore, this first set of growth curves is meant to align with CMAM reporting standards [46]. Another set of growth curves inclusive of all children was generated to assess overall trends in the study dataset. The overall pattern of growth was visualized using aggregate growth curves. Median MUAC or WAZ at admission and at each subsequent visit up to and including discharge were estimated using a non-parametric percentile bootstrap procedure with 10,000 bootstrap replicates. These estimates were plotted, and a summary growth curve fitted using a LOWESS smoother [47] that weighted the weekly median estimates by the square root of the sample size at each week in each group. Summary growth curves were made for groups 1–3 for both the combined and standard protocol programs.

## 3. Results

**Figure 1:**
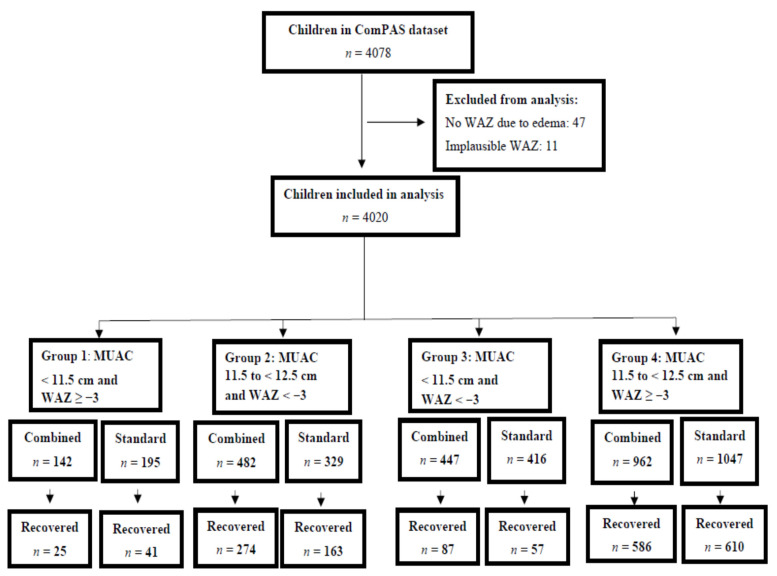
Analysis flow chart. MUAC, mid-upper arm circumference; WAZ, weight-for-age z-score.

Of the 4078 children included in the ComPAS trial dataset [35], 58 were excluded. Of these, 47 did not have an admission WAZ calculated due to the presence of oedema (which would have biased weight and WAZ upwards), and 11 were excluded because they had implausible WAZ values [44,45]. A total of 4020 children were available for the outcome analyses. The growth curve analyses presented in Figure 1 included the 647 children in groups 1–3 who recovered within 17 weeks. Of these 4020 children, 1962 were in Kenya, and 780 (39.8%) returned for a four-month follow-up visit, of whom 225 were in groups 1–3 (Supplementary Table S3).

Half of the children in this analysis were either severely underweight (WAZ < −3) and/or severely wasted (MUAC < 11.5 cm) at admission. Of the 4020 children, 1674 (41.6%) had a WAZ < −3. Of the 1200 children with MUAC < 11.5 cm, 71.9% had a WAZ < −3. Among those with a WAZ < −3863, 51.6% had a MUAC < 11.5 cm, and 811 (48.4%) had a MUAC ≥ 11.5 cm. Of the 2820 children with MUAC ≥ 11.5 cm, 28.8% had a WAZ < −3. Twice as many children in South Sudan (66.9%) were either severely underweight and/or severely wasted than in Kenya (32.4%). A total of 21.5% of children were both severely underweight by weight-for-age and severely wasted by MUAC (Table 2).

Overall, 1150 (89.5%) of children with a WHZ < −2.0 and HAZ < −2.0 (i.e., concurrently wasted and stunted) also had a WAZ < −3.0. In Kenya, fewer children with a WHZ < −2.0 and HAZ < −2.0 also had a WAZ < −3.0 (80.9%). In South Sudan, more children with a WHZ < −2.0 and HAZ < −2.0 also had a WAZ < −3.0 (93.3%) (Table 3).

Group 1 were younger (median age 10 months) than the other groups and had the highest proportion of females. Group 2 were the oldest (median age 20 months), and Group 3 had a median age of 15 months (Table 4).

In general, children with a MUAC < 11.5 cm (groups 1 and 3) had lower recovery and higher defaulting and non-response than children with a MUAC between 11.5 and <12.5 cm (groups 2 and 4) at admission. Within MUAC categories, severely underweight children had poorer outcomes than those without severe underweight. Of the three patient groups of interest, children in group 2 had the highest recovery (53.9%) and shortest length of stay (median 64 days), though recovery was lower and length of stay longer than for children in the comparator group 4 who had neither WAZ < −3.0 nor MUAC < 11.5 cm (of whom 59.5% recovered with a median length of stay of 57 days). Recovery in group 1 was 19.6%, and this group had the longest median length of stay (94 days). Children in group 3 had the lowest recovery (16.7%), despite being 5 months older on average than group 1. Children in group 3 also had the highest proportion of defaulters (39.4%) and deaths (1.9%). Defaulting and non-recovery was high in all groups studied [35], though group 2 had lower non-recovery (8.5%) and defaulting (21.7%) than groups 1 or 3. Among children with a MUAC between 11.5 cm and <12.5 cm, those with a WAZ < −3.0 had lower recovery (Table 5)

In exploratory analyses of the sub-groups by type of protocol given, there was no evidence of a difference between the combined and the standard protocols for groups 1 or 2 for any of the outcomes (Supplementary Tables S4–S6). There was weak evidence of a difference by type of protocol given in group 3 in terms of recovery and defaulting. Recovery in group 3 was 19.5% in the combined protocol compared to 13.7% in the standard protocol (adjusted risk ratio = 1.36, 95% CI 0.90, 1.82, *p* = 0.07). Defaulting in group 3 was 33.6% in the combined protocol compared to 45.2% in the standard protocol (adjusted risk ratio = 0.74, 95% CI 0.57, 0.97, *p* = 0.03) (Supplementary Table S6). There was no evidence of a difference in mortality for children when treated with either the combined or standard protocol in any of the three groups (Supplementary Tables S4–S6).

Relapse to acute malnutrition at the four-month post-discharge follow-up visit in Kenya among children who were discharged as recovered in the three patient groups (*n* = 142) ranged from 8% to 25%. A high proportion of children who were admitted with MUAC < 11.5 cm and/or oedema reported an illness in the week before their post-discharge follow-up appointment (20–47%). Body composition (lean and fat mass) was similar for those treated with the combined and the standard protocol in each of the admission groups (Supplementary Table S3).

**Figure 2:**
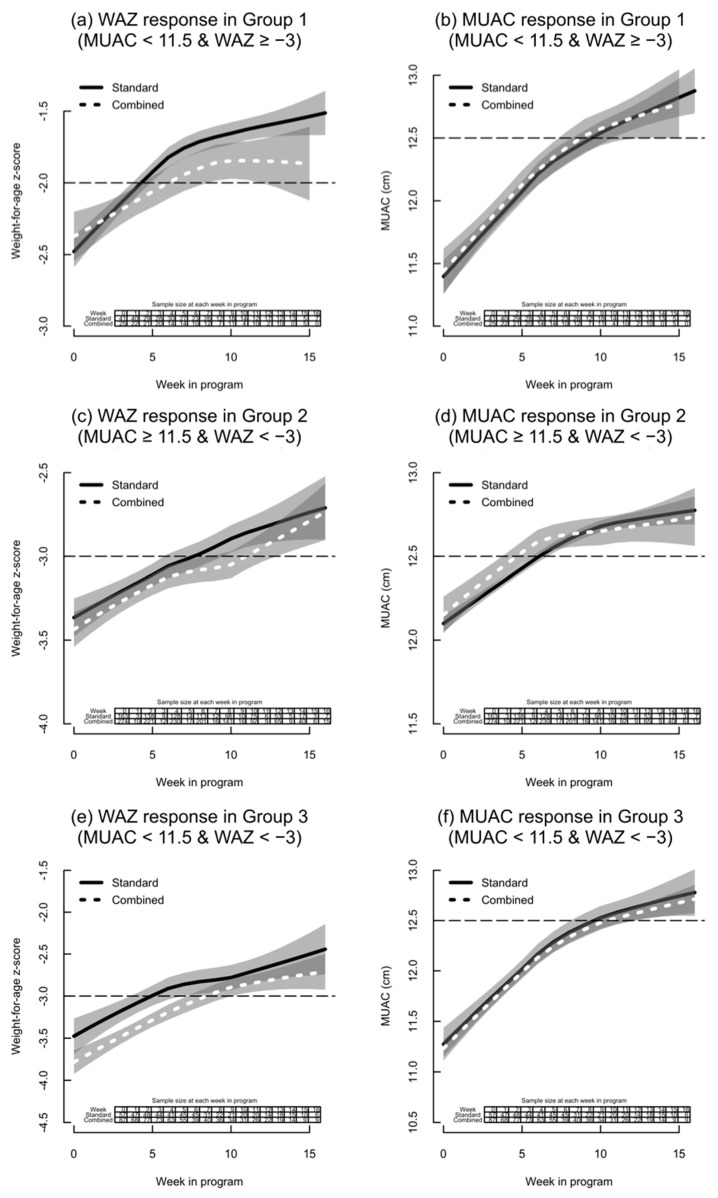
Panel of WAZ and MUAC response among recovered children by admission group. MUAC, mid-upper arm circumference; WAZ, weight-for-age z-score. (**a**) WAZ plotted against week in program for Group 1; (**b**) MUAC plotted against week in program for Group 1; (**c**) WAZ plotted against week in program for Group 2; (**d**) MUAC plotted against week in program for Group 2; (**e**) WAZ plotted against week in program for Group 3; (**f**) MUAC plotted against week in program for Group 3. The shaded areas represent a 95% confidence band around each curve (i.e., the area between the upper and lower 95% confidence limits is shaded). Overlaps between confidence intervals are more darkly shaded.

Growth curve analyses were conducted among recovered children in the three patient groups of main interest (Figure 2) and among all children (Supplementary Figure S1). The samples at each time point in the growth curves are small, and the 95% confidence bands between the combined and standard protocols mostly overlap, though there are some potential differences to highlight. Of children who recovered, WAZ gain appears slower and lower in Group 1 among children treated with the combined protocol (Figure 2a). There is no evidence of a difference in MUAC gain in this group. In Group 3, children in the combined protocol start out at a lower baseline for WAZ (Table 3), which may explain some of the difference in Figure 2e. As with Group 1, there is no evidence of a difference in MUAC gain between the protocols in Group 3 (Figure 2f). In Group 2, the 95% confidence bands overlap for both WAZ and MUAC gain with no significant differences between the protocols.

Similar trends can be seen in the growth curves of all children, with lower WAZ gain in children treated with the combined protocol in Group 1 (Supplementary Figure S1a), though 95% confidence bands overlap at many time points, and there is no evidence of a difference in MUAC gain (Supplementary Figure S1b). In Group 2, the children in the combined protocol appear to start at a lower baseline and the overall shape, and trajectory of WAZ gain is similar to the standard protocol, with mostly overlapping 95% confidence bands (Supplementary Figure S1c). There is no evidence of a difference in MUAC gain between the protocols in Group 2 (Supplementary Figure S1d). In Group 3, children in the combined protocol start at a lower baseline WAZ (Table 3), and the curve remains lower at all time points (Supplementary Figure S1e). MUAC gain appears slower and lower among children in the combined protocol in Group 3 (Supplementary Figure S1f).

**Figure 3:**
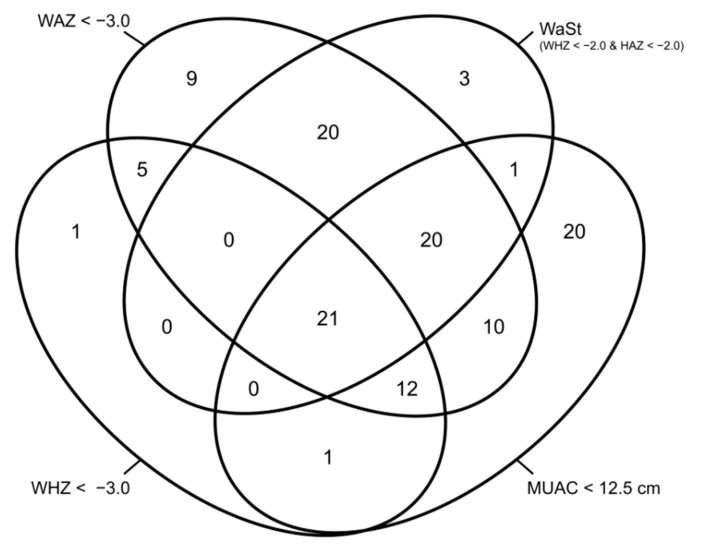
Venn diagram showing the number of deaths among treated children with different combinations of anthropometric deficits (adapted from Myatt et al., 2019 [19]). MUAC, mid-upper arm circumference; WAZ, weight-for-age z-score; WHZ, weight-for-height z-score; HAZ, height-for-age z-score; WaSt, both wasted and stunted.

To assess how well a combination of WAZ < −3.0 and MUAC < 12.5 cm performs in capturing near-term deaths (i.e., within 6 months), we adapted an analysis of data presented in a previous paper [19] to assess a cut-off of WAZ < −3.0 instead of WAZ < −2.8 (Figure 3). A combination of MUAC and WAZ case definitions detect 39 (97.5%) of the 40 deaths associated with a WHZ < −3.0 and 62 (95.4%) of 65 deaths associated with concurrent wasting and stunting, as defined by WHZ < −2.0 and HAZ < −2.0 (WaSt).

## 4. Discussion

Children with a MUAC between 11.5 cm and <12.5 cm who would be admitted to therapeutic feeding programs if WAZ < −3.0 is added as an independent criterion had higher recovery and lower lengths of stay than either of the groups with a MUAC < 11.5 cm, despite receiving only a supplementary dose of RUTF or RUSF instead of a therapeutic dose based on weight. Recovery in this group remained lower than children with the same MUAC (between 11.5 to <12.5 cm) but a higher WAZ (i.e., WAZ ≥ −3.0), which may reflect the increased severity of their anthropometric deficits. Children who recovered in this group also achieved rapid growth under both the combined and standard protocols, suggesting we may expect good response to treatment with 2092 kJ (500 kcal)/day of either RUTF or RUSF. Children with severely low MUAC (<11.5 cm), with and without severe underweight (WAZ < −3.0), had low recovery by 17 weeks (Table 5). These children also had the longest lengths of stay and the highest defaulting. Children with both a MUAC < 11.5 cm and a WAZ < −3.0 had the lowest recovery of any patient group, with the greatest number of deaths. There was no evidence of a difference in mortality for children when treated with either the combined or standard protocol in any of the three groups (Supplementary Tables S4–S6). This analysis indicates some potential differences in the way severely malnourished children respond to treatment between the combined and standard protocols, though these differences were mixed. There was weak evidence of higher recovery and lower defaulting among children with a MUAC < 11.5 cm and a WAZ < −3.0 treated with the combined protocol. Among children with a MUAC < 11.5 cm and a WAZ ≥ −3.0, there was some indication that WAZ gain was slower and lower in the combined protocol, though MUAC gain was similar. These mixed results in children with the most severe deficits in MUAC and WAZ warrant further investigation to understand the growth patterns and optimal dosage protocol for these groups.

Limitations to this analysis included both statistical and operational constraints. Caution should be exercised when extrapolating these results as these are exploratory analyses of small sub-groups. This is a secondary analysis of data from a trial that was not powered for sub-group analyses but for combined multi-country estimates of overall recovery in each treatment arm. The majority of children in the ComPAS trial were aged 6–24 months, thus limiting generalizability to older children. The eligibility criteria for this trial were focused on MUAC and/or oedema; therefore, we cannot assess how WAZ would perform as an independent criterion if we had admitted all those with WAZ < −3 but normal MUAC and oedema status. Operationally, the ComPAS trial faced multiple challenges. Study operations were disrupted by a nurse’s strike and repeated national elections in Kenya in 2017, and the rainy season interfered with the accessibility of some clinic sites in South Sudan [35]. These operational factors affected both study arms equally and contributed to frequent missed visits, longer lengths of stay, and higher numbers of children discharged as non-responders or defaulters. An outcome of “non-response” (defined as non-recovered in this analysis) was strictly applied to all children still in treatment after the cut-off of 17 weeks, though some of these children went on to recover after 17 weeks [35]. Low overall recovery was reported in the intention-to-treat analyses of the ComPAS trial due to high defaulting, though the main per-protocol analysis reported higher recovery than the intention-to-treat analysis (76.3% in the combined protocol and 73.5% in the standard arm) [35]. The high defaulting reduced the number of recovered children that could be analyzed in each of the three admissions groups in this analysis.

Strengths of this analysis included the multi-country nature of the trial, which contributes to the generalizability of results. Both rural and urban settings are included. Additionally, the data analyzed comes from a randomized controlled trial, with quality control measures in place to ensure high accuracy of data. The ComPAS trial provided a unique opportunity to evaluate WAZ response in different settings and in children with both SAM and MAM in the same dataset and to explore their outcomes by intensity of dosage and type of food product given [27].

This analysis contributes to a growing body of evidence on the utility of adding WAZ as an admission criterion for therapeutic feeding programs alongside MUAC and oedema [17,19,48]. As humanitarian and development actors and governments study the impacts of combined and simplified SAM and MAM programming [27,28,29,30,31,32,33,34,35,36], debate continues over which anthropometric indicators and cut-offs are likely to admit the highest-risk children that can be treated successfully with currently available therapeutic feeding products [21,49]. Given that a combination of low WAZ and/or low MUAC captures nearly all near-term mortality associated with anthropometric deficits including low WHZ and concurrent wasting and stunting, their combined use is an inclusive approach to therapeutic feeding admissions (Figure 3) [17,19]. In addition to being good predictors of near-term mortality, WAZ and MUAC are practical to use [19,22,23,50]. It is difficult to identify children with concurrent wasting and stunting and prioritize them for treatment because HAZ is not an admission criterion for therapeutic feeding. WAZ may be a more feasible community-based alternative. Community-level programs can use WAZ to identify children with a severely low WHZ who are at high risk of mortality without needing to measure height [17,19]. WAZ and MUAC are already widely used in community programs (such as Expanded Program on Immunization (EPI), Integrated Management of Childhood Illnesses (IMCI), Growth Monitoring and Promotion (GMP), and community screening). WAZ integrates multiple anthropometric dimensions of malnutrition, thus bridging the current divide in programming between wasting and stunting. The use of WAZ and MUAC also aligns with recent recommendations for the identification of SAM in infants less than 6 months [51].

These potential benefits must be weighed against potential drawbacks, including the difficulties assessing age in feeding programs and the likely increase in caseload. Age may be challenging to assess objectively, as it may be based on reporting using seasonal or cultural event calendars. This may affect the ability to accurately assess WAZ for some children. Current treatment programs, particularly those including moderate wasting, may already capture many low weight-for-age cases (41.6% of children admitted in the ComPAS trial had a WAZ < −3.0) (Table 2). Based on the numbers in this analysis, if WAZ < −3.0 (with a MUAC < 12.5 cm) is added as a criterion to a CMAM program admitting children with a MUAC < 11.5 cm only, the caseload would increase by 1.68 times (in this program, from 1200 children with a MUAC < 11.5 cm to 2011 children with either a MUAC < 11.5 cm and/or a WAZ < −3.0). This is similar to caseload change estimates made from simple simulations using population, prevalence survey, and program coverage data reported previously [19]. However, the exploratory analyses presented in this paper indicate this additional group of children appear to respond well to 1 sachet of either RUTF or RUSF a day and a clinic visit every other week instead of weekly, thus making this a potentially cost-effective approach. In emergencies or resource-constrained settings, either RUTF or RUSF could be selected based on availability. If this group of children identified by WAZ < −3 is added into a program using a simplified and combined protocol, additional cost savings may be seen. In this analysis, out of the 589 children with a MUAC < 11.5 cm (groups 1 and 3) treated with the combined protocol, 535 (90.8%) had an admission weight of ≥5 kg and thus received a reduced dosage of RUTF compared to standard care (Supplementary Table S2). Our prior research on this dosage protocol indicated it is sufficient to meet the theoretical energy needs of children 6–59 months recovering from uncomplicated acute malnutrition [34], and recovery was non-inferior to the standard protocol in an RCT [35]. The reduced dosage and other cost savings seen in a simplified protocol [30,35,36] may make it more feasible to treat additional children identified using WAZ and maintain the overall cost-effectiveness of the program.

Future work should evaluate how to treat children with the highest risk of mortality efficiently and effectively. This includes prospectively testing appropriate treatment regimens for the severely low MUAC and WAZ groups, assessing how adding WAZ to the admission criteria for therapeutic feeding affects caseload, resource use and workload, and evaluating outcomes of low weight-for-age and concurrently wasted and stunted children under treatment. Existing CMAM cohort data could be used for this purpose, as many SAM cases are likely to have a WAZ < −3.0 (Table 2). Future work could look at whether children who do not ultimately reach recovery criteria nonetheless improve their WAZ and MUAC. Different WAZ and MUAC cut-offs for admission and management can be tested according to context and in consideration of aggravating factors such as food insecurity and prevalence of disease. A WAZ < −3.0 identified different proportions of children with concurrent wasting and stunting in Kenya and South Sudan in this analysis (Table 3). Studies should build in adequate follow-up time post-treatment to assess longer-term growth trends and health outcomes of children with a low WAZ.

## 5. Conclusions

Preventing mortality and achieving optimal long-term health outcomes should be the goal of therapeutic feeding. This requires a more flexible view of what defines malnutrition to avoid the silos created by SAM/MAM or wasted/stunted classifications. Adding WAZ as an admissions criterion may help programs target children at higher risk of adverse outcomes who may benefit from treatment. The optimal dosage protocol for the most severely malnourished (MUAC < 11.5 cm, with and without a WAZ < −3.0) should be evaluated further.

## Figures and Tables

**Table 1:** Energy and food product given by treatment group.

Group	Combined Protocol	Standard Protocol
**Group 1:** MUAC < 11.5 cm and WAZ ≥ −3.0	4184 kJ (1000 kcal)/day RUTF	836.8 kJ (200 kcal)/kg/day RUTF
**Group 2:** MUAC 11.5 to <12.5 and WAZ < −3.0	2092 kJ (500 kcal)/day RUTF	2092 kJ (500 kcal)/day RUSF
**Group 3:** MUAC < 11.5 cm and WAZ < −3.0	4184 kJ (1000 kcal)/day RUTF	836.8 kJ (200 kcal)/kg/day RUTF
**Group 4:** MUAC 11.5 to <12.5 and WAZ ≥ −3.0	2092 kJ (500 kcal)/day RUTF	2092 kJ (500 kcal)/day RUSF

MUAC, mid-upper arm circumference; WAZ, weight-for-age z-score; RUTF, ready-to-use-therapeutic food; RUSF, ready-to-use supplementary food.

**Table 2:** Numbers of children by category at admission.

Admission Groups	Total by Category	By Intervention Arm		By Country	
		Combined Protocol	Standard Protocol	Kenya	South Sudan
**Group 1** MUAC < 11.5 cm and WAZ ≥ −3.0	337 (8.4%)	142 (7%)	195 (9.8%)	126 (6.4%)	211 (10.3%)
**Group 2** MUAC 11.5 to <12.5 cm and WAZ < −3.0	811 (20.2%)	482 (23.7%)	329 (16.6%)	294 (15%)	517 (25.1%)
**Group 3** MUAC < 11.5 cm and WAZ < −3.0	863 (21.5%)	447 (22%)	416 (20.9%)	215 (11%)	648 (31.5%)
**Group 4** MUAC 11.5 to <12.5 cm and WAZ ≥ −3.0	2009 (50%)	962 (47.3%)	1047 (52.7%)	1327 (67.6%)	682 (33.1%)
Total	4020	2033	1987	1962	2058

MUAC, mid-upper arm circumference; WAZ, weight-for-age z-score.

**Table 3:** Numbers of children at admission with a WHZ < −2.0 and HAZ < −2.0 identified by a WAZ < −3.0.

Country	Criterion	WAZ < −3.0	WAZ ≥ −3.0	Total
Kenya	WHZ < −2 and HAZ < −2	322 (80.9%)	76 (19.1%)	398
South Sudan	WHZ < −2 and HAZ < −2	828 (93.3%)	59 (6.7%)	887
Both	WHZ < −2 and HAZ < −2	1150 (89.5%)	135 (10.5%)	1285

WHZ, weight-for-height z-score; HAZ, height-for-age z-score; WAZ, weight-for-age z-score.

**Table 4:** Admission characteristics for the three patient groups of interest, by protocol type.

Characteristic	Group 1 MUAC < 11.5 cm and WAZ ≥ −3.0 (*n* = 337)		Group 2 MUAC 11.5 to <12.5 cm and WAZ < −3.0 (*n* = 811)		Group 3 MUAC < 11.5 cm and WAZ < −3.0 (*n* = 863)	
	Combined (m ^§^ = 12, *n* ^†^ = 142)	Standard (m = 12, *n* = 195)	Combined (m = 12, *n* = 482)	Standard (m = 12, *n* = 329)	Combined (m = 12, *n* = 447)	Standard (m = 12, *n* = 416)
Sex and Age						
Males, *n* (%)	31 (21.8%)	45 (23.1%)	258 (53.5%)	194 (59%)	221 (49.4%)	196 (47.1%)
Age at admission (months), median (IQR)	10 (8, 14)	10 (7, 14)	18 (12, 30)	21 (14, 30)	14 (9, 24)	15 (10, 24)
Age 6–24 months, *n* (%)	119 (83.8%)	168 (86.2%)	274 (56.8%)	173 (52.6%)	306 (68.5%)	287 (69.0%)
**Anthropometry**						
Weight (kg), mean (SD)	7.00 (1.54)	6.97 (1.62)	7.30 (1.42)	7.52 (1.39)	6.39 (1.34)	6.50 (1.31)
Height (cm) *, mean (SD)	72.6 (9.28)	72.0 (9.29)	74.0 (8.34)	74.9 (8.06)	70.8 (8.07)	71.5 (8.09)
MUAC (cm), mean (SD)	11.2 (0.23)	11.2 (0.29)	12.0 (0.27)	12.0 (0.26)	11.0 (0.44)	11.0 (0.49)
WAZ, mean (SD)	−2.4 (0.56)	−2.4 (0.52)	−3.6 (0.47)	−3.6 (0.52)	−4.1 (0.67)	−3.9 (0.66)
HAZ, mean (SD)	−0.91 (1.07)	−0.99 (1.16)	−3.05 (1.02)	−3.07 (1.03)	−3.13 (1.25)	−2.82 (1.17)
WHZ, mean (SD)	−2.61 (1.01)	−2.52 (0.73)	−2.72 (0.79)	−2.62 (0.81)	−3.23 (0.83)	−3.24 (0.86)

MUAC, mid-upper arm circumference; WAZ, weight-for-age z-score; HAZ, height-for-age z-score; WHZ, weight-for-height z-score.^§^ m = number of clusters; ^†^ *n* = individual children eligible for treatment. * Length if child is <24 months.

**Table 5:** Outcomes of children, by admission category.

	MUAC < 11.5 cm				MUAC 11.5 to <12.5 cm			
Intention-to-Treat	Group 1 MUAC < 11.5 cm and WAZ ≥ −3.0 (*n* = 337)		Group 3 MUAC < 11.5 cm and WAZ < −3.0 (*n* = 863)		Group 2 MUAC 11.5 to <12.5 cm and WAZ < −3.0 (*n* = 811)		Group 4 MUAC 11.5 to <12.5 cm and WAZ ≥ −3.0 (*n* = 2009)	
	*n*	%	*n*	%	*n*	%	*n*	%
Recovered *	66	19.6	144	16.7	437	53.9	1196	59.5
**Died**	3	0.9	16	1.9	7	0.9	18	0.9
**Defaulted**	131	38.9	340	39.4	176	21.7	480	23.9
**Non-recovered** ^†^	102	30.3	225	26.1	69	8.5	176	8.8
**Transfer-inpatient**	9	2.7	29	3.4	10	1.2	8	0.4
**Transfer-new facility**	8	2.4	33	3.8	15	1.9	13	0.7
**Early discharge**	18	5.3	78	9.0	102	12.6	130	6.5
	**Median**	**IQR**	**Median**	**IQR**	**Median**	**IQR**	**Median**	**IQR**
**Length of stay (days)** ^‡^	94	73, 106	89.5	68, 101	64	43, 85	57	43, 76

MUAC, mid-upper arm circumference; WAZ, weight-for-age z-score.* Recovery is defined as MUAC ≥ 12.5 cm and no oedema for 2 consecutive visits. ^†^ Non-recovered is defined as not reaching recovery criteria after 17 weeks in treatment, even though some of these children achieved recovery after 17 weeks [35]. ^‡^ Length of stay among recovered children only, following global CMAM reporting standards [46].

## Data Availability

Underlying data and code for this paper are available via https://datacompass.lshtm.ac.uk/1151/. The full dataset cannot be made open access due to the presence of participant identifiable content. A redacted version will be provided to interested parties, subject to the completion of a request form (available via the repository link above) and signing of a Data Transfer Agreement.

## Supplementary Materials

The following are available online at https://www.mdpi.com/2072-6643/13/4/1054/s1. Table S1. Comparison of combined and standard protocols. Table S2. Standard ready-to-use therapeutic food dosage table (based on 200 kcal/kg/day using 92 g packets containing 500 kcal). Table S3. Relapse, body composition, and morbidity at four months post-discharge, by admission category and protocol type (Kenya sample only). Table S4. Outcomes of children in group 1, unadjusted and adjusted risk ratios. Table S5. Outcomes of children in group 2, unadjusted and adjusted risk ratios. Table S6. Outcomes of children in group 3, unadjusted and adjusted risk ratios. Figure S1. Panel of WAZ and MUAC response among all children by admission group.
