# Supplementary material for: Response to Malnutrition Treatment in Low Weight-for-Age Children: Secondary Analyses of Children 6–59 Months in the ComPAS Cluster Randomized Controlled Trial
Source: Nutrients. 2021 Mar 24;13(4):1054. doi: 10.3390/nu13041054 (PMC8064102; doi:10.3390/nu13041054)
Supplement: Supplementary file 1 [file nutrients-13-01054-s001.pdf]

Supplementary Table S1. Comparison of Combined and Standard Protocols *

	**Standard Protocol (CONTROL)**	**Combined Protocol (INTERVENTION)**
**Eligibility criteria**	Age 6–59 months, MUAC < 12.5 cm and/or oedema (+/++), and clinically uncomplicated (i.e., passes appetite test, no Integrated Management of Childhood Illness (IMCI [†])**Error! Bookmark not defined.** danger signs/ no serious medical complications)	Age 6–59 months, MUAC <12.5cm and/or oedema (+/++), and clinically uncomplicated (i.e., passes appetite test, no Integrated Management of Childhood Illness (IMCI) danger signs/ no serious medical complications)
**Admission criteria**	**OTP**<br>• WHZ < −3<br>AND/OR<br>• MUAC < 11.5 cm<br>AND/OR<br>• Bilateral pitting oedema (+/++)<br>AND<br>• clinically uncomplicated	• <12.5 cm MUAC<br>AND/OR<br>• Bilateral pitting oedema (+/++)<br>AND<br>• clinically uncomplicated
	**SFP**<br>• Discharged from OTP<br>AND/OR<br>• WHZ < −2.0 to WHZ > −3<br>AND/OR<br>• MUAC ≥ 11.5 cm and < 12.5 cm<br>AND<br>• clinically uncomplicated	
**Treatment frequency**	**OTP**	**MUAC <11.5 cm and/or edema (+/++)**
	Weekly	Weekly
	**SFP**	**MUAC ≥ 11.5 and < 12.5cm**
	14 days	14 days
**Treatment transition criteria**	• Child meets OTP 'recovered' definition as described below	• Two consecutive MUAC measurements at or above 11.5 cm<br>AND<br>• No edema
**Dosage**	**OTP**	**MUAC < 11.5 cm and/or edema (+/++)**
	RUTF 200 kcal/kg/day	RUTF 1000 kcal/day (2 sachets/day)
	**SFP**	**MUAC ≥ 11.5 and < 12.5 cm**
	RUSF 500 kcal/day (1 sachet/day)	RUTF 500 kcal/day (1 sachet/day)
**Recovered**	**OTP**<br>• Child maintains MUAC ≥ 11.5 cm for two consecutive visits<br>AND/OR<br>• WHZ > −3 z-score for two consecutive visits<br>AND<br>• No edema for two consecutive visits	≥ 12.5 cm for 2 consecutive measurements and no edema
	**SFP**<br>Child maintains WHZ > −2.0 z-score and/or MUAC ≥ 12.5 cm for a period of two consecutive visits	

MUAC, mid-upper arm circumference; IMCI, Integrated Management of Childhood Illness; WHZ, weight-for-height z-score; OTP, outpatient therapeutic program; SFP, supplementary feeding program; RUTF, ready-to-use therapeutic food; RUSF, ready-to-use supplementary food. * Adapted from Bailey et al. 2018 [27] and Bailey et al. 2020 [35]. † Integrated Management of Childhood Illness (IMCI) (revised). 2014. http://www.who.int/maternal_child_adolescent/documents/IMCI_chartbooklet/en/ (accessed on 22 March 2021).

Supplementary Table S2. Standard ready-to-use therapeutic food dosage table (based on 200 kcal/kg/day using 92 g packets containing 500 kcal) *

Child's Weight (kg)	Packets per Day	Packets per Week
4.0 [†]–4.9	2	14
5.0–6.9	2.5	18
7.0–8.4	3	21
8.5–9.4	3.5	25
9.5–10.4	4	28
10.5–11.9	4.5	32
≥12	5	35

* Adapted from Bailey et al 2020 [35]. [†] Infants ≥ 6 months and < 4kg are referred to in-patient care.

Supplementary Table S3. Relapse, body composition and morbidity at four months post-discharge, by admission category and protocol type (Kenya sample only)

**Intention-to-Treat**	**Group 1** MUAC < 11.5 cm and WAZ ≥ −3.0 (*n* = 29)				**Group 2** MUAC 11.5 to < 12.5 cm and WAZ < −3.0 (*n* = 140)				**Group 3** MUAC < 11.5 cm and WAZ < −3.0 (*n* = 56)			
	**Combined** (*N* = 6, *n* = 10)		**Standard** (*N* = 6, *n* = 19)		**Combined** (*N* = 6, *n* = 87)		**Standard** (*N* = 6, *n* = 53)		**Combined** (*N* = 6, *n* = 30)		**Standard** (*N* = 6, *n* = 26)	
	n	%	***n***	%	***n***	%	***n***	%	***n***	%	***n***	%
**Acute malnutrition status** †	2	20	5	26	15	17	10	19	7	23	5	19
**Relapse to acute malnutrition** ‡)	1/4	25	1/10	10	10/68	15	6/35	17	1/12	8	1/13	8
**Illness reported in past week** §	2	20	9	47	21	24	12	23	6	20	8	31
**Hospitalization reported in past 4 months**	0	0	1	5	5	6	3	6	1	3	2	8
	mean	SD	mean	SD	mean	SD	mean	SD	mean	SD	mean	SD
**Fat free mass (kg)**	6.29	0.54	6.26	0.62	6.08	0.82	6.22	0.88	6.18	0.66	6.16	0.70
**Fat mass (kg)**	2.26	0.61	2.43	0.63	2.15	0.84	2.31	0.93	2.44	1.14	2.10	0.77

MUAC, mid-upper arm circumference; WAZ, weight-for-age z-score. *N* = number of clusters; *n* = individual children eligible for treatment. † At 4-month follow-up visit. ‡ Among those discharged as cured. § Data collected on reported diarrhea, vomiting, fever or cough in past week.

Supplementary Table S4. Outcomes of children in group 1, unadjusted and adjusted risk ratios

Intention-to-Treat	Group 1 MUAC < 11.5 cm and WAZ ≥ −3.0 (*n* = 337)							
	Combined (*N* = 12, *n* = 142)		Standard (*N* = 12, *n* = 195)		Unadjusted Risk Ratio †		Adjusted Risk Ratio †	
	**n**	**%**	**n**	**%**	**Risk ratio (95% CI)**	***p*-Value ‡**	**Risk ratio (95% CI)**	***p*-Value ‡**
**Recovered**	25	17.6	41	21.0	0.84 (0.52, 1.35)	0.47	0.87 (0.54, 1.40)	0.56
**Died**	1	0.7	2	1.0	0.69 (0.07, 6.45)	0.74	0.57 (0.05, 6.55)	0.65
**Defaulted**	48	33.8	83	42.6	0.79 (0.57, 1.11)	0.18	0.81 (0.58, 1.12)	0.20
**Non-recovered** §	46	32.4	56	28.7	1.13 (0.75, 1.70)	0.57	1.16 (0.77, 1.74)	0.48
**Transfer-inpatient**	7	4.9	2	1.0	3.20 (0.68, 15.04)	0.14	3.13 (0.76–12.96)	0.12
**Transfer-new facility**	6	4.2	2	1.0	4.12 (0.74, 22.87)	0.11	4.08 (0.75, 22.13)	0.10
**Early discharge**	9	6.3	9	4.6	1.53 (0.61, 3.80)	0.36	1.54 (0.68, 3.45)	0.30
	**Median**	**IQR**	**Median**	**IQR**	**Common Language Effect Size statistic (control > intervention) * (95% CI)**			
**Length of stay (days)** a	80	59, 94	94	85, 108	0.63 (0.49, 0.78)			

MUAC, mid-upper arm circumference; WAZ, weight-for-age z-score. *N* = number of clusters; *n* = individual children eligible for treatment. † Both unadjusted and adjusted results account for the effect of clustering. The adjusted model also includes adjustment for country, age and sex. ‡ Analyses use the Generalized Linear Model, reporting Pearson chi-squared *p*-values. § Non-recovered defined as not reaching recovery criteria after 17 weeks in treatment. a Length of stay among recovered children only, per global CMAM reporting standards [46]. * Probability that a randomly selected control length of stay is greater than a randomly selected intervention length of stay.

Supplementary Table S5. Outcomes of children in group 2, unadjusted and adjusted risk ratios

Intention-to-Treat	Group 2 MUAC 11.5 to <12.5cm and WAZ < −3.0 (n=811)							
	Combined (N = 12, n = 482)		Standard (N = 12, n = 329)		Unadjusted Risk Ratio [†]		Adjusted Risk Ratio [†]	
	n	%	*n*	%	Risk Ratio (95% CI)	*p*-Value [‡]	Risk Ratio (95% CI)	*p*-Value [‡]
**Recovered**	274	56.9	163	49.5	1.15 (0.85, 1.55)	0.37	1.18 (0.90, 1.54)	0.24
**Died**	4	0.83	3	0.91	0.91 (0.16, 5.25)	0.92	0.75 (0.15, 3.86)	0.73
**Defaulted**	92	19.1	79	24.0	0.78 (0.45, 1.37)	0.39	0.78 (0.51, 1.19)	0.25
**Non-recovered** [§]	41	8.5	28	8.5	1.0 (0.40, 2.50)	1.0	0.82 (0.41, 1.63)	0.58
**Transfer-inpatient**	5	1.0	5	1.5	0.68 (0.13, 3.59)	0.65	0.68 (0.14, 3.28)	0.63
**Transfer-new facility**	9	1.9	6	1.8	0.98 (0.37, 2.60)	0.96	1.06 (0.45, 2.48)	0.90
**Early discharge**	57	11.8	45	13.7	0.89 (0.46, 1.72)	0.73	0.95 (0.57, 1.61)	0.86
	**Median**	**IQR**	**Median**	**IQR**	**Common Language Effect Size Statistic (control > intervention) * (95% CI)**			
**Length of stay (days)** [a]	57	43, 85	71	57, 85	0.52 (0.46, 0.58)			

MUAC, mid-upper arm circumference; WAZ, weight-for-age z-score. N = number of clusters; n = individual children eligible for treatment. [†] Both unadjusted and adjusted results account for the effect of clustering. The adjusted model also includes adjustment for country, age and sex. [‡] Analyses use the Generalized Linear Model, reporting Pearson chi-squared *p*-Values. [§] Non-recovered defined as not reaching recovery criteria after 17 weeks in treatment. [a] Length of stay among recovered children only, per global CMAM reporting standards [46]. *Probability that a randomly selected control length of stay is greater than a randomly selected intervention length of stay.

Supplementary Table S6. Outcomes of children in group 3, unadjusted and adjusted risk ratios

Intention-to-Treat	Group 3 MUAC < 11.5 cm and WAZ < −3.0 (*n* = 863)							
	Combined (*N* = 12, *n* = 447)		Standard (*N* = 12, *n* = 416)		Unadjusted Risk Ratio †		Adjusted Risk Ratio †	
	n	%	*n*	%	Risk ratio (95% CI)	*p*-Value ‡	Risk ratio (95% CI)	*p*-Value ‡
**Recovered**	87	19.5	57	13.7	1.42 (0.89, 1.95)	0.06	1.36 (0.90, 1.82)	0.07
**Died**	9	2.0	7	1.7	1.20 (0.30, 4.78)	0.25	0.91 (0.36, 2.33)	0.85
**Defaulted**	150	33.6	188	45.2	0.74 (0.56, 0.98)	0.03	0.74 (0.57, 0.97)	0.03
**Non-recovered** §	111	24.8	114	27.4	0.90 (0.70, 1.15)	0.40	0.91 (0.73, 1.15)	0.45
**Transfer-inpatient**	18	4.0	11	2.6	1.40 (0.34, 5.69)	0.47	1.58 (0.38, 6.56)	0.53
**Transfer-new facility**	21	4.7	12	2.9	1.43 (0.64, 3.17)	0.38	1.51 (0.68, 3.35)	0.31
**Early discharge**	51	11.4	27	6.5	1.80 (0.95, 3.40)	0.07	1.95 (1.10, 3.43)	0.02
	**Median**	**IQR**	**Median**	**IQR**	**Common Language Effect Size statistic (control > intervention) * (95% CI)**			
**Length of stay (days)** [a]	81	66, 101	94	71, 108	0.56 (0.46, 0.67)			

MUAC, mid-upper arm circumference; WAZ, weight-for-age z-score. *N* = number of clusters; *n* = individual children eligible for treatment. †Both unadjusted and adjusted results account for the effect of clustering. The adjusted model also includes adjustment for country, age and sex. ‡ Analyses use the Generalized Linear Model, reporting Pearson chi-squared *p*-values. § Non-recovered defined as not reaching recovery criteria after 17 weeks in treatment. [a] Length of stay among recovered children only, per global CMAM reporting standards [46]. * Probability that a randomly selected control length of stay is greater than a randomly selected intervention length of stay.

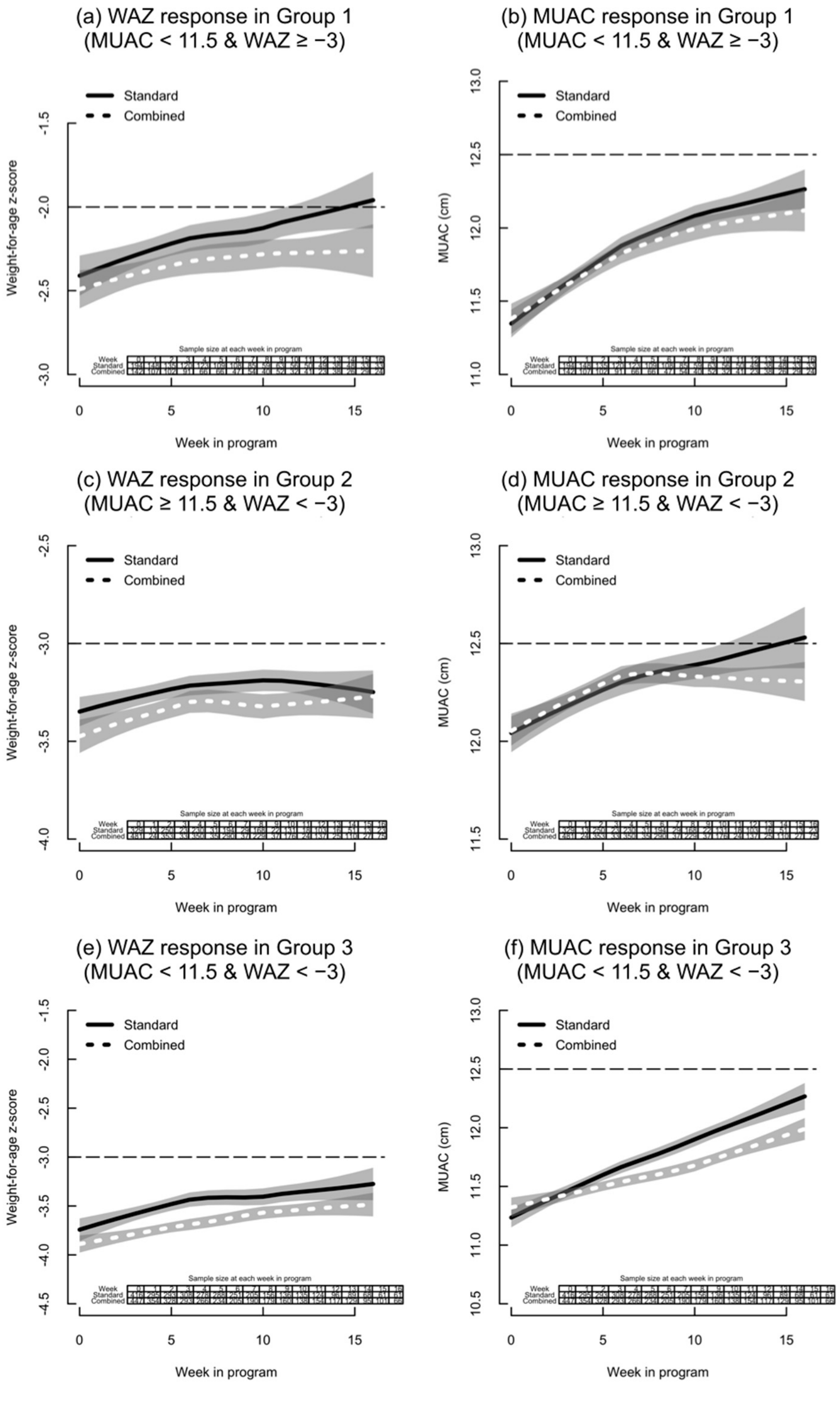

Supplementary Figure S1. Panel of WAZ and MUAC response among all children by admission group. MUAC, mid-upper arm circumference; WAZ, weight-for-age z-score. (**a**) WAZ plotted against week in program for Group 1; (**b**) MUAC plotted against week in program for Group 1; (**c**) WAZ plotted against week in program for Group 2; (**d**) MUAC plotted against week in program for Group 2; (**e**) WAZ plotted against week in program for Group 3; (**f**) MUAC plotted against week in program for Group 3. The shaded areas represent a 95% confidence band around each curve (i.e., the area between the upper and lower 95% confidence limits is shaded). Overlaps between confidence intervals are more darkly shaded.
